# Indole-3-lactic acid associated with *Bifidobacterium*-dominated microbiota significantly decreases inflammation in intestinal epithelial cells

**DOI:** 10.1186/s12866-020-02023-y

**Published:** 2020-11-23

**Authors:** Amy M. Ehrlich, Alline R. Pacheco, Bethany M. Henrick, Diana Taft, Gege Xu, M. Nazmul Huda, Darya Mishchuk, Michael L. Goodson, Carolyn Slupsky, Daniela Barile, Carlito B. Lebrilla, Charles B. Stephensen, David A. Mills, Helen E. Raybould

**Affiliations:** 1 Department of Anatomy, Physiology and Cell Biology, School of Veterinary Medicine, University of California, Davis, CA 95616 USA; 2 Foods for Health Institute, University of California, Davis, CA USA; 3 Department of Food Science and Technology, University of CA, Davis, CA USA; 4 Department of Chemistry, University of California, Davis, CA USA; 5 Enteric and Respiratory Infections Unit, Infectious Diseases Division, icddr,b, Dhaka, Bangladesh; 6 US Department of Agriculture, Western Human Nutrition Research Center, Davis, CA USA; 7 Department of Nutrition, University of California, Davis, CA USA

**Keywords:** Milk oligosaccharides, Aryl-hydrocarbon receptor, Nuclear factor erythroid 2–related factor 2, Indole-3-lactic acid

## Abstract

**Background:**

*Bifidobacterium longum* subsp. *infantis* (*B. infantis*) is a commensal bacterium that colonizes the gastrointestinal tract of breast-fed infants. *B. infantis* can efficiently utilize the abundant supply of oligosaccharides found in human milk (HMO) to help establish residence. We hypothesized that metabolites from *B. infantis* grown on HMO produce a beneficial effect on the host.

**Results:**

In a previous study, we demonstrated that *B. infantis* routinely dominated the fecal microbiota of a breast fed Bangladeshi infant cohort (1). Characterization of the fecal metabolome of binned samples representing high and low *B. infantis* populations from this cohort revealed higher amounts of the tryptophan metabolite indole-3-lactic acid (ILA) in feces with high levels of *B. infantis*. Further in vitro analysis confirmed that *B. infantis* produced significantly greater quantities of the ILA when grown on HMO versus lactose, suggesting a growth substrate relationship to ILA production. The direct effects of ILA were assessed in a macrophage cell line and intestinal epithelial cell lines. ILA (1-10 mM) significantly attenuated lipopolysaccharide (LPS)-induced activation of NF-kB in macrophages. ILA significantly attenuated TNF-α- and LPS-induced increase in the pro-inflammatory cytokine IL-8 in intestinal epithelial cells. ILA increased mRNA expression of the aryl hydrogen receptor (AhR)-target gene CYP1A1 and nuclear factor erythroid 2–related factor 2 (Nrf2)-targeted genes glutathione reductase 2 (GPX2), superoxide dismutase 2 (SOD2), and NAD(P) H dehydrogenase (NQO1). Pretreatment with either the AhR antagonist or Nrf-2 antagonist inhibited the response of ILA on downstream effectors.

**Conclusions:**

These findings suggest that ILA, a predominant metabolite from *B. infantis* grown on HMO and elevated in infant stool high in *B. infantis*, and protects gut epithelial cells in culture via activation of the AhR and Nrf2 pathway.

**Supplementary Information:**

The online version contains supplementary material available at 10.1186/s12866-020-02023-y.

## Background

Numerous studies have correlated breastfeeding with significant decreases in infant morbidity and mortality. Breast milk provides high levels of innate and adaptive immune factors that have direct antibacterial and antiviral activity [1], however, it also provides high levels of free sugars known as human milk oligosaccharides (HMOs) [2]. HMOs make up the third largest component of breast milk yet are indigestible by enzymes present in the infant gut. HMOs likely have many functions; they can mimic receptor sites for pathogenic microbes in the gut [3, 4] and have direct effects on intestinal epithelial cells [4, 5]. However, HMOs also provide the growth substrate for beneficial commensal bacteria, such as *Bifidobacterium* species that are equipped with the cellular machinery to breakdown and utilize these complex substrates as an energy source [2, 6–8].

*Bifidobacterium longum* subsp. *infantis* (*B. infantis*) is an important early colonizer and one of the predominant species in the intestinal microbiome of breastfed infants [9, 10]. *B. infantis* expresses an impressive repertoire of oligosaccharide transporters and glycosyl hydrolases required to consume complex sugars internally [11, 12] which provides a growth advantage [13]. A recent study demonstrated how supplemented *B. infantis* can dominate the gut of breast-fed infants, consume HMOs and dramatically modulate the colonic pH via production of acetic and lactic acids [14]. Other studies have suggested *B. infantis* to be beneficial to the infant in numerous ways, including providing antimicrobial properties, improving intestinal permeability and reducing inflammation in the gut [15–20].

In addition to any direct effects that *B. infantis* or HMOs have on the host, there is good evidence to suggest that bacterial metabolites can also have beneficial effects on host physiology. Various metabolites, including short chain fatty acids (SCFA), phenylalanine metabolites and tryptophan metabolites have been shown to have broadly beneficial effects on host health [21, 22]. We have previously shown that direct interaction of *B. infantis* grown on HMOs with intestinal epithelial cells significantly reduced markers of inflammation by comparison to *B. infantis* grown on either glucose or lactose [17]. This suggests that the carbon source changes the composition of bioactive metabolites secreted from *B. infantis*. However, the precise identity of the metabolites mediating these effects and the mechanism of their action on the intestinal mucosa remains largely unknown. We hypothesized that growth of *B. infantis* on HMOs compared to simple carbohydrates such as lactose, results in secretion of bioactive metabolites that influence the gastrointestinal tract of the host. Therefore, to identify the effect of carbon source on metabolite composition, *B. infantis* was grown either on HMOs or lactose as the sole carbon source and NMR was used to identify metabolites. We sought to identify the mechanism by which a particular metabolite, indole-3-lactic acid (ILA), that was enriched when *B. infantis* was grown on HMOs, inhibits release of inflammatory cytokines in intestinal epithelial cells in vitro. Further, we determined the pathway by which ILA acts, showing activation of the aryl hydrocarbon receptor (AhR) and nuclear factor erythroid 2–related factor 2 (Nrf2)-targeted genes. Finally, the concentration of ILA and short chain fatty acids (SCFAs) was measured in fecal samples from breastfed infants that had a predominantly *Bifidobacterium*-dominated intestinal microbiome. The data show that ILA is in high concentration in the feces of infants with a *Bifidobacterium*-dominated intestinal microbiome, is an enriched metabolite of *B. infantis* when grown of HMOs and produces marked anti-inflammatory effects via activation of the ArhR pathway.

## Results

### Indole-3-lactic acid is elevated in infant fecal samples with a high compared to low abundance of *B. infantis*

**Fig. 1:**
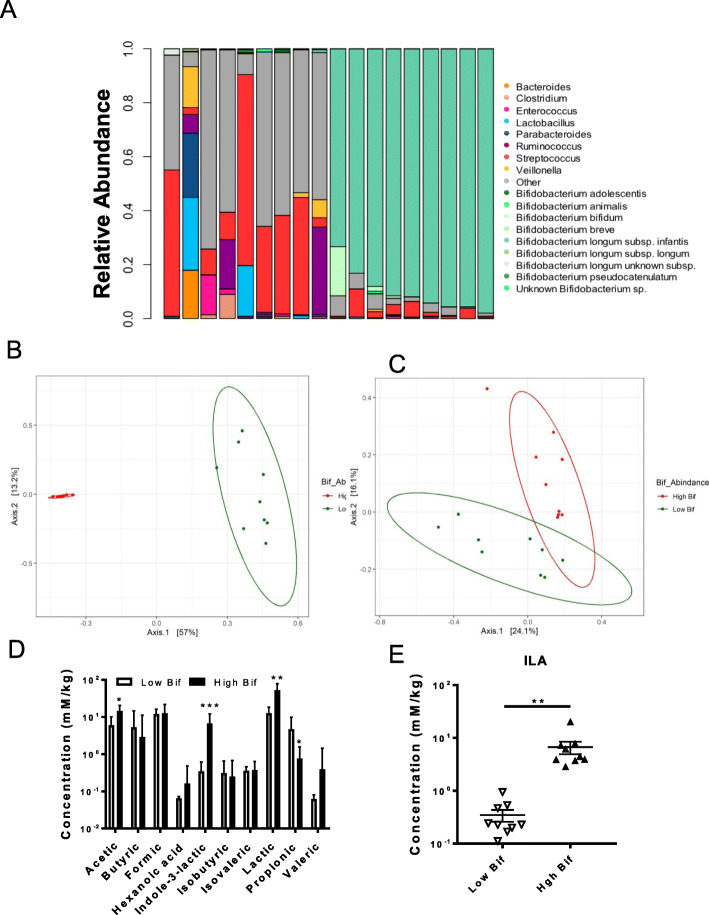
Increased ILA production in fecal samples from infants with high levels of bifidobacteria. Determination of microbiota in infant fecal samples using 16S, Bif-TRFLP, and BLIR assays. **a** Fecal samples showed low and high distributions of *Bifidobacterium* among breast fed infants (*n* = 18) previously characterized (1). In fecal samples with a high level of *Bifidobacterium,* the distribution of species and subspecies is shown in different shades of green (note *B. longum* subsp. *infantis* dominating all of the high *Bifidobacterium* feces). **b** PCoA plots of weighted and **c** unweighted β diversity was significantly different between high and low Bifidobacterium groups. **d** Metabolite analyses of infant fecal samples by LC-MS/MS (72). **e** High *Bifidobacterium* samples showed highly significant differences in ILA production (*p* < 0.0001) compared to low *Bifidobacteriu*m samples. Student t-test was used to determine significance with corresponding *P* values considered statistically significant in *p < 0.05, **p < 0.01, *** *p =* 0.0001

Previously, we characterized a cohort of Bangladeshi breast fed infants revealing a high (dominating) level of *B. infantis* occurring frequently among this cohort (1, 23). To examine if this high bifidobacterial content was associated with specific metabolic products, we binned fecal samples for high and low bifidobacterial content and performed mass spectrometry-based metabolomics. Fecal samples were selected based on total *Bifidobacterium* and *B. infantis* relative abundance with 9 samples containing the highest abundance and 9 with the lowest abundance [23]. The low *Bifidobacterium* samples contained an average of 0.96% *Bifidobacterium* (median 1%; standard deviation of 0.7%), and will be referred to as ‘low *Bifidobacterium”* group. The high *Bifidobacterium* samples had an average of 93.56% (median 92%; standard deviation 2.55%) *Bifidobacterium* and will be referred to as ‘high *Bifidobacterium’* group. In this high *Bifidobacterium* group, the next most abundant genera were represented by *Streptococcus*, *Lactobacillus* and *Enterococcus*. Conversely, the ‘low *Bifidobacterium’* fecal samples were populated with various genera, including predominantly *Streptococcus* and *Enterococcus* genera (Fig. 1a). In the samples with high *Bifidobacterium* abundance, the mean relative abundance of *B. infantis* represented 97.22% (median 1.0; standard deviation 6.53%) from the total *Bifidobacterium* population. The gut microbial weighted (betadisper: F.model = 4.96, *P* = 0.048; ADONIS: F.model = 34.0, R2 = 0.680, *P* < 0.01) and unweighted (betadisper: F.model = 0.446, *P* = 0.50; ADONIS: F.model = 2.87, R2 = 0.152, *P* < 0.01) β diversity was significantly different between high and low Bifidobacterium groups (Fig. 1b and c).

Short chain fatty acids and the indole derivative ILA were measured in the 18 infant fecal samples using mass spectrometry. The concentration of ILA was significantly elevated in fecal samples from infants with a high versus low *Bifidobacterium*-dominated microbiome (Fig. 1d, e; *P <* 0.0001). The *B. infantis*-dominated microbiome population produced a greater than 19-fold increase in ILA concentration compared to the low-*Bifidobacterium* dominated infant microbiome (Fig. 1d, e;). The concentration of acetic and lactic acid was significantly increased in the fecal samples from the high versus low *Bifidobacterium* groups (Fig. 1d; *P =* 0.0353, *P =* 0.0095, respectively). There was also a significant decrease in the concentration of propionic acid in the high versus low *Bifidobacterium* groups (*P* = 0.049). There was no significant difference in the concentrations of the other detectable SCFA (butyric, formic, isobutyric, and valeric) between the high versus low bifidobacterial groups.

### Growth on HMOs versus lactose determines *B. infantis* metabolite profile in vitro

The observation of increased ILA from *B. infantis*-dominated feces suggested this metabolite emanated from *B. infantis*. One mechanism by which *B. infantis* dominates the infant gut is through the consumption of complex human milk oligosaccharides (HMOs) in milk (15). Production of acetate, lactate and formate by *Bifidobacterium* species has previously been shown to be dependent on the carbon source in the growth medium [24] but how growth on HMOs influences production of metabolites from *B. infantis* has not been described. In order to determine the differences in the metabolites produced by *B. infantis* grown on two different carbon sources, we performed untargeted metabolomic analysis of the supernatant from *B. infantis* grown in chemically defined RPMI 1640 medium supplemented with either lactose or HMO. *B. infantis* grown on HMO or lactose produced a large number of metabolites, including amino acids, organic acids and carbohydrates (Supplemental Fig. 1). The data show significantly increased production of acetate, glutamate, glycerol, glycine, indole-3-lactate, leucine, pyruvate, and valine in HMO-supplemented compared to lactose-supplemented medium (Supplemental Fig. 1; all *P <* 0.0001). HMO-supplemented growth of *B. infantis* produced greater than 4-fold more ILA compared to bacteria grown in lactose-supplemented media (Supplemental Fig. 1, insert; *P* < 0.0001), suggesting that production of ILA is increased when *B. infantis* is consuming milk glycans.

**Fig. 2:**
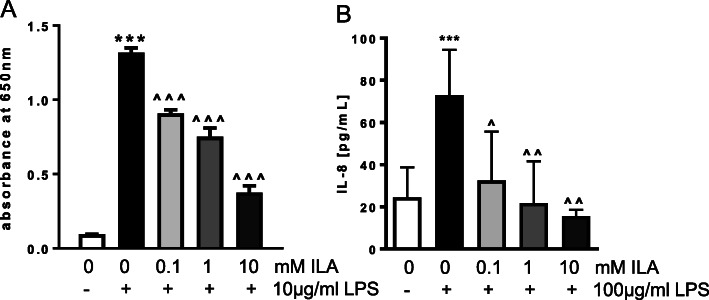
ILA inhibits LPS-induced inflammation in macrophage and intestinal epithelial cells. **a** SEAP activity in RAW blue macrophage cells exposed to media alone, LPS alone, or with different concentrations of ILA for 1 h prior to addition of LPS. **b** IL-8 from IECs exposed to media alone, LPS alone for 18 h or with different concentrations of ILA for 1 h prior to LP. Data is expressed as mean ± SEM, *n* = 3–7 and Student t-test was used to determine significance with corresponding *P* values considered statistically significant; *** *p <* 0.0001 compared to no treatment control or ^ *p* < 0.05, ^^ *p* < 0.01, ^^^ *p* < 0.0001 compared to LPS alone

Recently, it was shown that supplementing the diet of weanling piglets with tryptophan decreased expression of inflammatory markers in the large intestine [25] which may be due to the increased production of tryptophan metabolites. ILA is a known catabolite of tryptophan [26] and ILA was significantly elevated when *B. infantis* was grown on HMOs and in the feces containing high levels of *B. infantis*. Therefore, we investigated whether addition of tryptophan would alter metabolite composition when *B. infantis* was grown in RPMI 1640 supplemented with either HMO or lactose in the presence of increasing concentrations of tryptophan. Metabolomics analysis revealed that tryptophan produced an increase in the production of glutamate, glycerol, ILA, leucine, and valine in HMO-supplemented compared to lactose-supplemented medium (Supplemental Fig. 1; *P =* 0.0001, *P =* 0.0077, *P =* 0.0009, *P =* 0.0001, *P =* 0.0040, respectively). Increasing the tryptophan also significantly increased the catabolism of 3’FL, 6′SL, cysteine, glucose and LNnT in HMO (Fig. 2: *P* = 0.0037; *P* < 0.0001; *P* = 0.0003; *P* < 0.0001; *P* < 0.0001, respectively).

Given the differential amounts of ILA between feces containing high versus low abundance of *B. infantis*, combined with the increase in ILA production from *B. infantis* grown on HMO, and its increase concentration in the presence of tryptophan in the growth medium, ILA appeared to be an appropriate target for further study. ILA production differs from the other significant metabolites that were also increased by growth on HMOs compared to lactose as it is not directly involved in energy metabolism. ILA and other tryptophan metabolites are known to produce beneficial effects in the host [27–29]. We hypothesized that ILA may be responsible, at least in part, for the anti-inflammatory effects associated with *B. infantis*.

### Indole-3-lactic acid decreases endotoxin-induced activation of macrophages, and release and expression of IL-8 in intestinal epithelial cells

To test for a potential anti-inflammatory role of ILA in the intestinal mucosa, studies were performed in a macrophage cell line and intestinal epithelial cell lines in vitro. Initial experiments to show a potential anti-inflammatory action was performed in the mouse macrophage reporter cell line RAW blue cells. Subsequent experiments were performed in the intestinal epithelial cell lines Caco2 and HT-29. Two different cell lines were used to ensure that the anti-inflammatory response to ILA could be reproduced in both cell lines. Experiments on dissecting the AhR and Nrf2 pathway were performed in HT-29 cells.

Given the central role NFκB plays in the activation of innate immune responses [30], we first evaluated the ability of ILA to modulate LPS-induced immune activation using the mouse macrophage reporter cell line RAW blue cells. Overnight treatment of the mouse RAW blue cells with LPS (10 μg/ml) significantly increased activation of NFκB (Fig. 2a, *P* < 0.0001); pretreatment with ILA (0.1 mM–10 mM) significantly inhibited LPS-induced NFκB activation in a dose-dependent manner (Fig. 2a; *P* < 0.0001). Similar anti-inflammatory effects were seen in Caco-2 cells; pretreatment with ILA significantly decreased LPS-induced production of IL-8 (Fig. 2b; *P =* 0.0006; *P* = 0.0021).

**Fig. 3:**
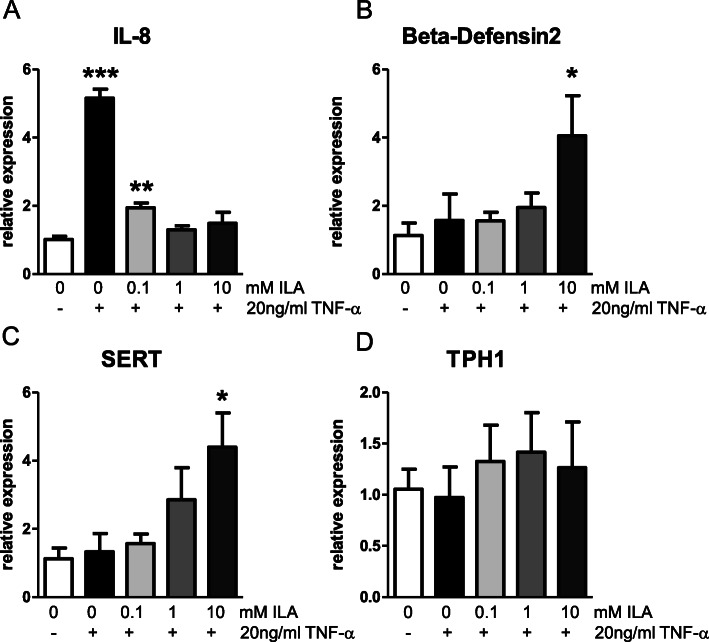
ILA inhibits TNF-α induced inflammation in intestinal epithelial cells. mRNA expression in IEC exposed to media alone, TNF-α or TNF-α in the presence of different concentrations of ILA for 1 h. **a** IL-8, **b** beta-defensin2, **c** SERT and **d** TPH1 mRNA expression. Data is expressed as mean ± SEM, *n* = 6 for each condition and Student t-test was used to determine significance with corresponding *P* values considered statistically significant (*p < 0.05, **p < 0.01, *** *p* < 0.0001)

**Fig. 4:**
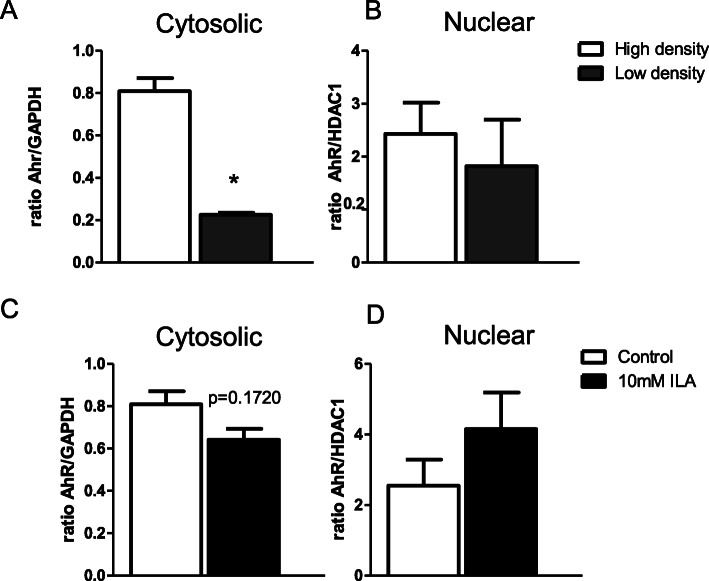
Cell density or presence of ILA affects AhR localization. **a** AhR cytosolic protein expression in IEC plated at high density (100% confluency) versus low confluency (50% confluency). **b** AhR nuclear protein expression in high and low density IECs (*p =* ns). **c** AhR cytosolic protein expression in high density IEC treated with ILA. **d** AhR nuclear protein expression in high density IEC treated with ILA*.* Protein was pooled from 6 individual plates of cells. Data is expressed as mean ± SEM; *n* = 2 and student t-test was used to determine significance with corresponding *P* values considered statistically significant in *p < 0.05, **p < 0.01, *** *p* < 0.0001

Similar anti-inflammatory actions were seen in HT-29 cells. LPS is a potent stimulus for release of the pro-inflammatory cytokine tumor necrosis factor-alpha (TNF-α) [31], therefore, we determined the ability of ILA to decrease inflammatory responses to TNF-α. HT-29 cells were incubated with TNF-α (20 ng/ml) for 1 h in the absence (control) or presence of ILA (0.1-10 mM). Cellular responses were analyzed using qPCR for IL-8 (immune activation), serotonin transporter (SERT) and tryptophan hydroxylase 1 (TPH1) (ILA is an indole derivative which have previously been shown to interact with serotonin receptors), and β-defensin 2 (hBD2; antimicrobial defense protein previously shown to be activated by bifidobacteria). TNF-α induced a robust increase in IL-8 mRNA expression (*P* < 0.0001) but there was no change in expression of SERT, hBD2, or TPH1 (Fig. 3). The increase in expression of IL-8 induced by TNF-α was significantly decreased by treatment with ILA (Fig. 3a; *P* < 0.0001 for 0.1, 1, and 10 mM ILA); there was no significant difference in expression of IL-8 between cells treated with TNF-α in the presence of ILA and those treated with vehicle alone (Fig. 3a, *P* = 0.061, *P* = 0.90 *P* = 0.59 for 0.1, 1, and 10 mM ILA, respectively). Further, expression of SERT and hBD2 were significantly increased in cells treated with ILA (10 mM) (Fig. 4b, c*, P* = 0.016 and *P* = 0.042). Expression of TPH-1 was not affected by treatment with TNF-α in the presence or absence of ILA (Fig. 3d).

### ILA activates the aryl hydrocarbon receptor (AhR) and downstream nuclear factor erythroid-derived 2-like 2 (Nrf2) pathway in intestinal epithelial cells

There is evidence that tryptophan catabolites contribute to gut homeostasis via modulating mucosal immune responses through an AhR signaling pathway [26, 32]. AhR is a nuclear receptor that when activated translocates to the nucleus to increase transcription of AhR-regulated genes. Previous work has indicated that AhR changes its cellular location based on cell density [33]. To confirm this occurs in intestinal cells, we measured protein expression of AhR by western blot in cytosolic and nuclear extracts from Caco-2 cell cultured at either low (50% confluency) or high (100% confluency). Consistent with published data from other cell types [28], intestinal epithelial cells grown at high density have increased cytosolic AhR compared to cells plated at low density (Fig. 4a, *P* = 0.01). For a preliminary test that ILA can activate AhR, AhR expression was measured in the nuclear and cytosolic fractions in Caco-2 cells grown at high density in response to ILA. There was a trend for increased AhR expression in the nucleus and decreased AhR expression in the cytosol, indicating that ILA may activate AhR (Fig. 4c, d).

**Fig. 5:**
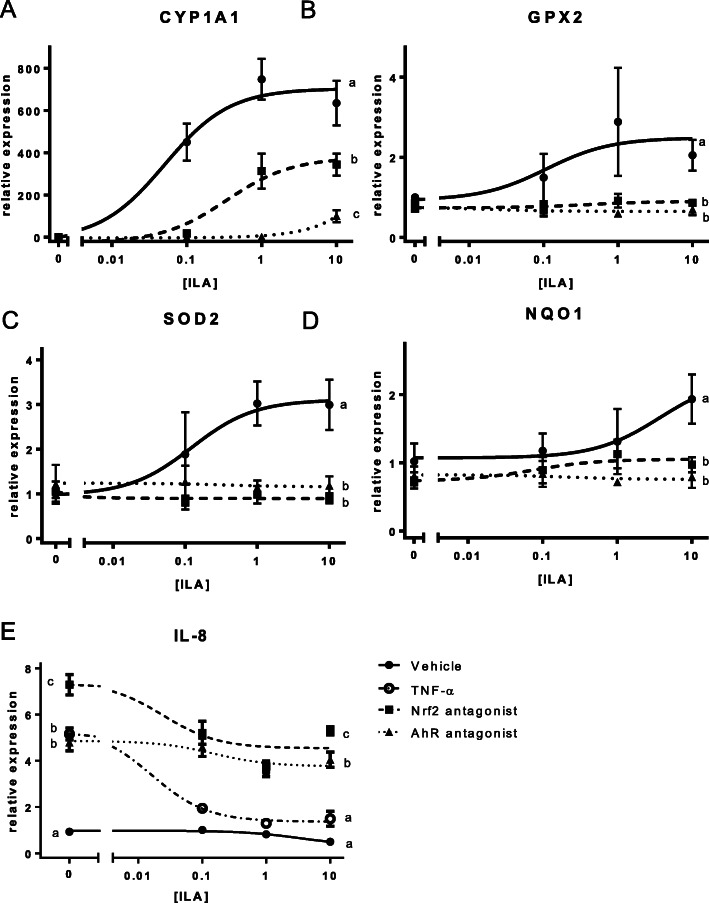
AhR or Nrf2 inhibition prevents ILA-induced response in intestinal epithelial cells. mRNA expression in IEC treated with ILA alone or with AhR or Nrf2 antagonist. **a** CYP1A1 **b** GPX2 **c** NQO1 **d** SOD2 **e** IL-8 mRNA. Data is expressed as mean ± SEM with *n* = 6–8 of relative expression values fitted with a 3-paramenter dose response curve vs the log-transformed ILA concentration (Prism, v. 8.0, GraphPad Software, San Diego, CA). Statistical significance of the effect of each treatment on the means of relative expression of each target gene at the highest ILA concentration (10 mM) was determined using a one-way ANOVA analysis with a TukeyHSD posthoc comparison (R, version 3.5.2 (1)). For IL-8, the statistical differences between the means in the absence of ILA was as also determined by this method. Different letters represent significantly different means. A summary of statistics can be found in Supplemental Table 3

In order to further understand the potential role of AhR in the anti-inflammatory response to AhR, we used HT-29 cells. Treatment of HT-29 cells with ILA (0.1–10 mM) induced a robust increase of mRNA transcript of AhR regulated gene cytochrome p450 1A1 (CYP1A1), which was significantly decreased by pretreatment with the AhR antagonist (CH-223191; 10 μM) (Fig. 5a, *P* = 0.0000003) or Nrf2 antagonist (SML1833; 4 μM) (Fig. 5a, *P* = 0.0001066). There is considerable evidence for cross talk between the AhR and Nrf2 pathways, which led us to test activation of the Nrf2 pathway [34]. Upon exposure to ILA, there were dose-dependent increases in Nrf2 activated genes glutathione peroxidase 2 (GPX2) and super oxide dismutase 2 (SOD2) and an increase in NAD(P) H dehydrogenase (NQO1) was observed. Inhibition of either AhR or Nrf2 completely suppresses ILA activation of GPX2, SOD2, and NQO1 genes (Fig. 5a-d, all *P* < 0.001).

To test if the AhR or Nrf2 pathway is involved in the ILA-induced suppression of pro-inflammatory IL-8 production, HT-29 cells were treated with TNF-α (20 ng/ml) and ILA (0.1-10 mM) in the presence of either an AhR antagonist or a Nrf2 inhibitor. ILA treatment produces a dose-dependent decrease in TNF-α-induced IL-8 expression which is inhibited by pretreatment with either an AhR antagonist or a Nrf2 inhibitor (Fig. 5e, all *P* < 0.0001).

## Discussion

Development of the microbiome starts at birth and there is evidence for the importance of a *Bifidobacterium*-dominated microbiome in breastfeeding infants to provide protection from inflammatory diseases [35, 36]. The enrichment of *B. infantis* in breastfed infants is believed to be driven by its ability to completely digest complex milk sugars (HMOs) leading to the conclusion that this subspecies is a keystone bacterium of the infant intestine [11, 12, 37]. Previous studies have reported on the anti-inflammatory effects of *B. infantis* both in vivo and in vitro [38, 39]. How *Bifidobacterium* are mediating their effects is not well understood but previous studies point to bioactive HMO metabolites produced by *Bifidobacterium*. Therefore, the purpose of the present study was to identify the metabolites produced by *B. infantis* that have anti-inflammatory effects and determine the pathways by which they act. Here we show, by comparing metabolites generated from *B. infantis* grown on HMO with specific metabolites found in the fecal samples from breast fed babies with a *B. infantis*-dominated microbiome, that ILA was significantly enriched in both sample sets. Further, our data shows that ILA significantly decreased production of the pro-inflammatory cytokine IL-8 in intestinal epithelial cell lines and decreased activation of macrophage cell line. In intestinal epithelial cells, the ability of ILA to decrease TNF-α-induced IL-8 expression was significantly reduced by pretreatment with either an AhR or Nrf2 antagonist and ILA treatment was associated with an increased expression of AhR and Nrf2 related genes. Taken together, these data show that a metabolite from *B. infantis,* which is both enriched during growth on HMOs as well as found in fecal samples from high-*Bifidobacterium* dominated infant fecal samples, can produce anti-inflammatory action via the AhR and Nrf2 pathway.

Initial work on AhR focused on its role in mediating the effects of toxic chlorinated dioxins and similar halogenated aromatic hydrocarbon compounds on many cell types and tissues [40], but it is evident that this receptor also plays a crucial role in regulating intestinal homeostasis. For example, AhR deficiency impairs intestinal stem cells from properly repairing after tissue damage [41]. Further, mice with dysregulated AhR regulated gene CYP1a1 mimicking an AhR-deficient state exhibited significantly decreased ability to clear infections from enteric pathogens [42]. Interestingly, when the AhR ligand indole-3-propionic acid (IPA) is administered to mice with DSS-induced colitis, there is reduction in pro-inflammatory cytokines and attenuation of disease severity. This is proposed to be the result of AhR mediated IL-10 induction [32]. There are a number of other downstream mediators that have been proposed, including production of anti-inflammatory cytokines such as IL-22 from intestinal immune cells, differentiation of CD4 + Foxp3 T cells, and more recently, increased expression of anti-inflammatory cytokine receptors [19, 32, 43, 44]. Numerous plant polyphenols such as polydatin, quercetin, reutin, and resveratrol [45], and an increasing list of metabolites produced from microbes such as IPA, indole-3-acetaldehyde, and indole-3-aldehyde [28], known to exert beneficial effects have been recently shown to act through AhR [29]. However, specific metabolites produced from *Bifidobacterium* are just recently beginning to be identified as anti-inflammatory mediators acting through AhR [46]. Blood samples from humans administered *B. infantis* have shown in increased secretion of IL-10 and expression of Foxp3 [47] suggesting that *B. infantis* leads to AhR activation though it is not clear whether it is a direct or indirect effect.

**Fig. 6:**
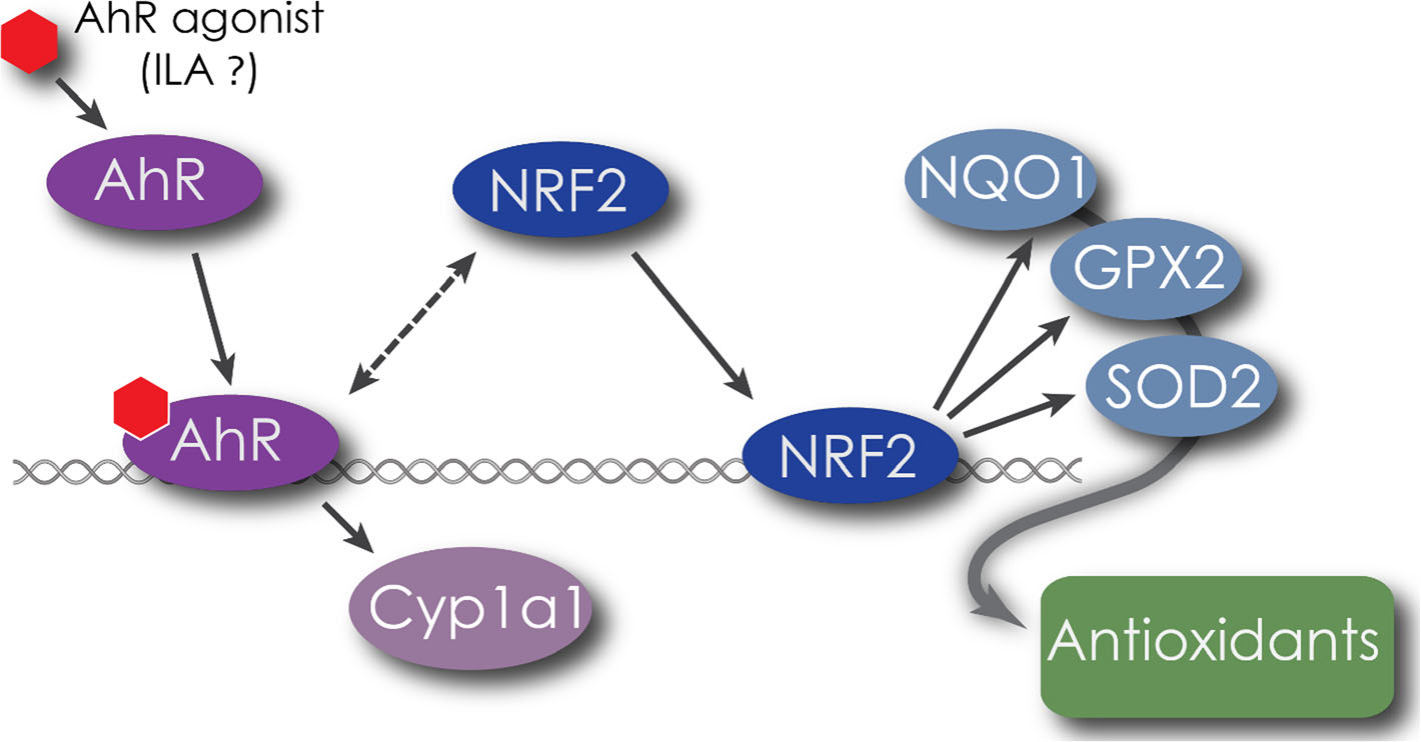
Proposed model of ILA activation. ILA activates AhR which increases expression of AhR regulated gene CYP1A1 as well as increasing activity of Nrf2. Activated Nrf2 leads to increased expression of cytoprotective genes to reduce oxidative cellular stress brought about by inflammatory agents such as LPS or TNF-α

Our data suggests additional beneficial effects of activating AhR are mediated through its cross talk with Nrf2 pathway. The Nrf2 pathway is associated with cytoprotective, redox sensitive pathways conferring resistance to oxidative stress [34, 48]. One particular Nrf2 agonist, sulforophane, has been shown to attenuate inflammation and disease in LPS-challenged mice [49] and in a chemically-induced model of diabetes [50] via similar mechanisms as we have observed in the current study, with ILA suppressing NF-kB activation and decreasing production of pro-inflammatory cytokines. The anti-inflammatory effects observed in this study are in corroboration with recent findings demonstrating protection against necrotizing enterocolitis with ILA through AhR [46] but here we provide evidence to also suggest a role for Nrf2. The potential interaction of ILA with these pathways is depicted in Fig. 6.

It has been previously demonstrated that ILA is produced by 51 species of *Bifidobacterium* incubated with tryptophan so it was not surprising to find increased ILA production with increasing concentrations of tryptophan [19]. High concentrations of tryptophan have been shown in human milk during the neonatal period [51]. Recent reports highlight the significance of tryptophan by showing anti-inflammatory effects in the colon of piglets ingesting a diet supplemented with tryptophan [25]. Thus, in order to evaluate whether the presence of additional tryptophan influences metabolite production by *B. infantis* grown on HMO, the concentration of tryptophan in growth media was increased. With increased tryptophan, we found increased production of ILA. In light of the observation that TNBS-induced colitis is increased in severity in SERT-deficient mice, we looked at whether the potential beneficial effects of the *B. infantis* metabolite ILA might also have effects on the serotonergic system [52, 53]. ILA had no effect on TPH1 expression, the enzyme that catalyzes the production of 5-HT from tryptophan, in intestinal epithelial cells. However, there was a significant upregulation of SERT; increased SERT could decrease inflammation by increasing transport of 5-HT away from the mucosa.

Similarly, ILA increases expression of human beta-defensin2 (hBD2). Lower levels of hBD2 have been found in patients with Crohn’s disease, perhaps due to a reduced ability to induce expression [54] or due to decreased number of copies of the hBD2 gene [55]. It does seem plausible that ILA could interact with the serotonergic system since particular residues in the 5-HT_1b_ binding pockets specifically bind the indole ring common to ILA and serotonin [56]. Additionally, it is possible ILA engages the vitamin D receptor to increase hBD2 expression. Previous reports have described cross-talk with NRF2 agonist sulforophane and the vitamin D receptor leading to induced expression of hBD2 through this transcription factor in intestinal cells in culture [57].

## Conclusion

In conclusion, these results identify significantly increased production of ILA by bifidobacterial-dominated breast fed infants. This is in line with previous publications that show ILA as a predominant tryptophan catabolite produced by many species in the *Bifidobacterium* genus in vitro [19]. Critically, we show the metabolite profile produced by *B. infantis* grown in vitro depends on growth on HMO compared to lactose, and production of the key metabolite ILA being significantly increased when HMO is available for *B. infantis*. The data shown here indicates that intestinal epithelial cells exposed to ILA produced significantly less pro-inflammatory cytokine in a dose-dependent manner providing a possible mechanism for the anti-inflammatory effects and improved intestinal health observed in *Bifidobacterium*-dominated breast fed babies. The observation that ILA activates AhR and Nrf2 and that this activation is required for ILA to regulate pro-inflammatory genes provides additional key mechanistic insight into how *Bifidobacterium* and HMO may be eliciting their anti-inflammatory effects to improve intestinal health. Together, these results have important implications for our fundamental understanding of metabolite production by specific bacterial species and support the notion that ILA might be considered an important regulator of intestinal inflammation in the breastfeeding infant. These, and other recent data may provide critical insight for generation of probiotics and dietary recommendations.

## Methods

### Infant fecal samples collection

We utilized 18 fecal samples (9 with highest and 9 with lowest *Bifidobacterium* abundance) originally collected as part of a larger trial in Bangladesh (clinicaltrials.gov identifier: NCT01583972 and NCT02027610). Detailed study design, sample collection procedures, and initial results have been reported earlier [23, 58–60]. Briefly, parents of infants born at the Maternal and Child Health Training Institute in Dhaka, Bangladesh were approached during the third trimester of pregnancy and informed consent was obtained. Infants were supplemented with a high dose vitamin A within 48 h of birth and stool samples were collected at 6 weeks of age and stored at − 70 C, as part of the original study. The study was approved by the Ethical Review Committee of the International Centre for Diarrheal Disease Research, Bangladesh (ICDDR,B) and by the Human Studies Committee of the World Health Organization.

### Infant gut microbiota assay

Whole stool samples were collected and were frozen at − 70 °C until assay. Detailed of the 16S assay including randomization, V4 amplicon library preparation, and bioinformatics analysis have been reported previously [59]. In brief, stool DNA samples were randomized, total fecal DNA was extracted and mixed template 16S V4 amplicon library was prepared using the primer sets (515F and barcoded 806R) [61] and sequenced using the Illumina MiSEQ platform with 2x250bp paired-end sequencing. Raw paired-end sequences were analyzed using the open-source software Quantitative Insights Into Microbial Ecology (QIIME) version 1.8 [62]. Taxonomy was assigned using the Greengenes May 2013 reference database [63] at the threshold of 97% pairwise identity. To estimate *Bifidobacterium* species and subspecies, we used Bif-TRFLP (*Bifidobacterium*-specific terminal restriction fragment length polymorphism) assay and BLIR (*Bifidobacterium longum* subsp. *longum-infantis* Ratio) assay, respectively, as has been reported earlier [23]. The relative abundance of the infant’s gut microbiota presented as combined results from 16S QIIME, Bif-TRFLP, and BLIR assay. The 16S sequence data is available at NCBI Sequence Read Archive (SRA) database under the BioProject ID “PRJNA636907” and “PRJNA636905”.

### Fecal metabolite analysis by LC-MS/MS

Pre-weighed fecal samples are diluted, homogenized, and centrifuged before extraction of the fecal supernatants. 20 μL of supernatant is reacted with 20 μL of 200 mM N-(3-Dimethylaminopropyl)-N′-ethylcarbodiimide hydrochloride (1-EDC HCl) in 5% pyridine and 40 μL of 100 mM 2-nitrophenylhydrazine (2-NPH) in 80% ACN/H_2_O (v/v) with 50 mM HCl. The samples were reacted for 30 min at 40 °C before being diluted with 400 μL of 10% ACN/H_2_O. Samples were centrifuged for 20 min before transferring to a 96-well injection plate for QqQ LC-MS/MS analysis. Dynamic MRM method was used in positive mode for detection and quantitation of the compounds. The quantification is absolute concentration and are shown as mmol/kg of original fecal sample wet weight [64].

### Bacterial growth and supernatant preparation

Single colony isolates of *B. infantis* ATCC 15697 were grown from frozen stocks on de Mann, Rogosa, and Sharpe medium (MRS) supplemented with 2% (wt/vol) carbon source and 0.25% (wt/vol) L-cysteine (Sigma-Aldrich) for 18 h, centrifuged for 10 min at 4000 rpm, resuspended in glucose-free RPMI 1640 (Life Sciences) then inoculated in three biological replicates at 1:100 ratio in RPMI 1640 (chemically defined) containing 2% of human milk oligosaccharides (HMO) previously purified in the UCD Milk Processing Lab or lactose (control). To obtain lactose-free HMOs, a purification approach of pooled donor milk based on lactose hydrolysis, yeast consumption of the resulting monosaccharides and further clean-up and concentration by sequential membrane filtration was used [65]. The freeze-dried powder was characterized by mass spectrometry and analyzed by high-performance anion-exchange chromatography with pulsed amperometric detection using a previously published method tailored for oligosaccharide quantification in milk [66] (Supplemental Table 1). *B. infantis* was incubated at 37 °C until early stationary phase under anaerobic conditions (5% carbon dioxide, 5% hydrogen, and 90% nitrogen) in a vinyl anaerobic chamber (Coy Laboratory Products). Bacterial growth was monitored by optical density (OD_600_) and an aliquot of the bacterial suspension was taken for determination of the bacterial counts by plating on MRS Agar. Sample preparation for exometabolome analysis was performed as described by Smart et al [67]*.* Cell-free supernatants were obtained by centrifuging the bacterial cultures at 4000 rpm for 15 min, followed by filtration using 0.22 μm filters (Millipore) to remove microbial cells and addition of 0.2 μmol per sample of isobutyric acid as internal standard. Samples were distributed in 100uL aliquots and stored at − 80 °C until processing. Uninoculated growth media was collected and used as controls for exometabolome analysis.

### NMR spectroscopy

Frozen supernatant samples were thawed and filtered through 3000 MW cutoff filters to remove lipids and proteins. Samples were prepared by addition of 65 μL of an internal standard containing 5 mM DSS-d6 (2,2,3,3,4,4-d6–3-(trimethylsilyl)-1-propane sulfonic acid), 0.2% NaN_3_ in 99% D_2_O to 585 μL of supernatant. Sample pH was subsequently adjusted to 6.8 through the addition of small amounts of NaOH or HCl. 600 μL aliquots were transferred to 5 mm Bruker NMR tubes, and stored at 4 °C until NMR acquisition which was within 24 h of sample preparation. NMR spectra were acquired as previously described by He et al. [68] using a Bruker Avance 600 MHz NMR equipped with a SampleJet autosampler using a NOESY-presaturation pulse sequence (noesypr) at 25 °C. Once acquired, all spectra were processed as described [68] using Chenomx NMR Suite v6.1 Processor (Chenomx Inc., Edmonton, Canada). Metabolite quantification was achieved using the 600-MHz library from Chenomx NMR Suite v6.1 Profiler, which uses the concentration of a known reference signal (in this case DSS) to determine the concentration of individual compounds. Metabolites were quantified in micro-molar (μM) units and exported from Chenomx for statistical analysis. Metabolite concentrations were normalized to bacterial CFU and were calculated relative to the concentration found in the growth media whereby a positive number indicates microbial production and a negative indicates consumption.

### Cell lines and reagents

RAW-Blue cells (InvioGen), a mouse macrophage cell line engineered with secreted embryonic alkaline phosphatase (SEAP) reporter system, was used to evaluate LPS-induced NFκB activation in the presence or absence of ILA in vitro. ILA was added to RAW-Blue cells for 1 h before being exposed to LPS. After incubating for 4 h, SEAP levels were quantified by developing supernatant with QUANTI-Blue substrate for 1 h and reading absorption at 620 nm as stated in the manufacturer’s instructions (InvioGen). Caco-2 and HT-29 cells were purchased from ATCC and grown to the specifications. To assess IL-8 production in response to LPS, Caco-2 cells were pre-incubated with ILA for 1 h before overnight exposure to LPS. For gene expression experiments, cells were incubated with ILA in the presence of TNF-α for 1 h or pretreated with AhR or Nrf2 inhibitors for 1 h before 1 h exposure to ILA. Experiments were performed for a total of 6–8 biological replicates. ILA, AhR antagonist CH-223191, Nrf2 antagonist SML1833, TNF-α and LPS 0111:B4 were purchased from Sigma. GAPDH, anti-rabbit HRP and anti-biotin HRP antibodies were purchased from Cell Signaling Technology. AhR antibody was purchased from Invitrogen. HDAC1 antibody was from Santa Cruz Biotechnologies.

### Immunoassays

ELISA Duoset was used to measure IL-8 levels in Caco-2 supernatants according to manufacturer’s instructions (R&D Systems).

### Western blot

Nuclear and cytosolic fractions were prepared as described previously [69]. Cell lysates were evaluated for total protein content using Bradford assay and overall concentration for each samples adjusted with deionized water and boiled with 2x Laemmli reducing buffer. Individual samples were resolved in SDS-PAGE as per standard protocol. Signals were developed and detected with appropriate antibodies and bands were analyzed using BioRad Imager. GAPDH and HDAC1 were used as internal loading controls for cytosolic and nuclear fractions.

### cDNA analysis

Total RNA was extracted from human Caco-2 or HT-29 using TRIzol reagent (Invitrogen) following the manufacturer’s instructions. RNA was reverse transcribed with Bio Rad iScript cDNA synthesis kit. cDNA template was combined with PowerUp SYBR Green Master Mix (ThermoFisher) and quantitative PCR was run using Applied Biosystems QuantStudio 6 Real-Time PCR System. Primers used to amplify were cytochrome p450 1a1, glutathione peroxidase 2, superoxide dismutase 2, NAD(P) H dehydrogenase, interleukin-8, beta-defensin2, serotonin transporter, tryptophan-hydroxylase 1, 60S acidic ribosomal protein P13A. Primer sequences are provided in Supplemental Table 2.

### Statistical analysis

Student’s t test or One-way ANOVA with Bonferroni Multiple comparison test was performed using GraphPad Prism. A *P* value of < 0.05 was considered statistically significant. For ILA dose curves, the mean ± SEM of relative expression values were fitted with a 3-paramenter dose response curve vs the log-transformed ILA concentration (Prism, v. 8.0, GraphPad Software, San Diego, CA). Statistical significance of the effect of each treatment on the means of relative expression of each target gene at the highest ILA concentration (10 mM) was determined using a one-way ANOVA analysis with a TukeyHSD posthoc comparison (R, version 3.5.2 [70]). For IL-8, the statistical differences between the means in the absence of ILA was as also determined by this method. Differences in the homogeneity of microbiota between high and low *Bifidobacterium* groups were determined by using the PERMDISP2 function of R Package Vegan [71] with 999 permutations. Differences in microbial community β-diversity were tested by ADONIS (perMANOVA) in the R Package Vegan. Principal coordinate (PCoA) analysis was carried out by PhyloSeq [72]. Correlation analysis was carried out by Spearman correlation. Statistical analyses were performed by using R version 3.5.2 [70] and Prism, v. 8.0 (GraphPad Software, San Diego, CA). Graphs were prepared by GGplot2 [73] and Prism, v. 8.0 (GraphPad Software, San Diego, CA).

## Supplementary Information

**Additional file 1: Supplemental Figure 1.** Metabolites produced by growth if *B. infantis* on lactose or HMO in the differing concentrations of tryptophan. **Supplemental Table 1.** Composition of milk oligosaccharide. **Supplemental Table 2.** Primer sequences. **Supplemental Table 3.** Summary of statistics from TukeyHSD posthoc comparisons of ILA dose responses.

## Data Availability

The datasets used and analyzed during the current study are available from the corresponding author on reasonable request.
