# Supplementary material for: Indole-3-lactic acid associated with Bifidobacterium-dominated microbiota significantly decreases inflammation in intestinal epithelial cells
Source: BMC Microbiol. 2020 Nov 23;20:357. doi: 10.1186/s12866-020-02023-y (PMC7681996; doi:10.1186/s12866-020-02023-y)

Supplemental Figure 1: Metabolites produced by growth if *B. infantis* on lactose or HMO in the differing concentrations of tryptophan.

**
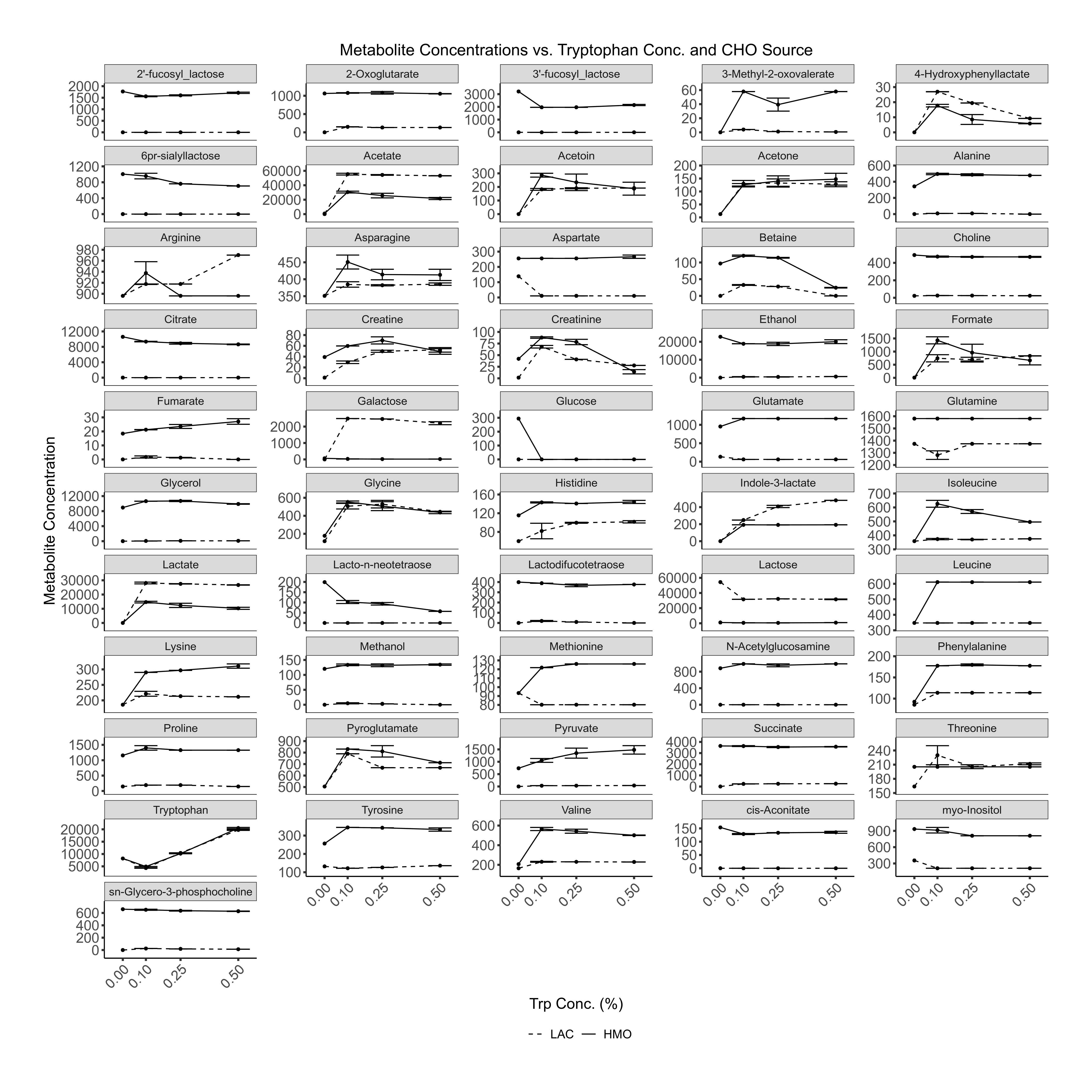
**

**Supplemental Table 1: Composition of milk oligosaccharide**

**Supplemental Table 2: Primer sequences**

**Supplemental Table 3: Summary of statistics from TukeyHSD posthoc comparisons of ILA dose responses**


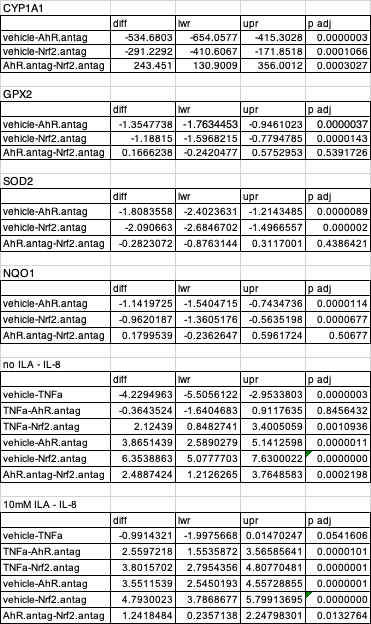

Supplement: Supplementary file 1 — Additional file 1: Supplemental Figure 1. Metabolites produced by growth if B. infantis on lactose or HMO in the differing concentrations of tryptophan. Supplemental Table 1. Composition of milk oligosaccharide. Supplemental Table 2. Primer sequences. Supplemental Table 3. Summary of statistics from TukeyHSD posthoc comparisons of ILA dose responses. [file 12866_2020_2023_MOESM1_ESM.docx]
